# Highly Sensitive and Selective Fluorescence “Turn-On” Detection of Pb (II) Based on Fe_3_O_4_@Au–FITC Nanocomposite

**DOI:** 10.3390/molecules26113180

**Published:** 2021-05-26

**Authors:** Yina Cai, Binxue Ren, Chifang Peng, Cunzheng Zhang, Xinlin Wei

**Affiliations:** 1 Jiangsu Key Laboratory for Food Quality and Safety-State Key Laboratory Cultivation Base, Ministry of Science and Technology, Nanjing 210014, China; 6200112045@stu.jaingnan.edu.cn; 2 Food Inspection and Quarantine Centre, Shenzhen Customs, Shenzhen 518045, China; 3 School of Food Science and Technology, Jiangnan University, Wuxi 214122, China; 6180112017@stu.jiangnan.edu.cn; 4 School of Agriculture and Biology, Shanghai Jiaotong University, Shanghai 200240, China; wxl@shnu.edu.cn

**Keywords:** gold nanoparticles, magnetic nanoparticles, FRET, nanomaterial etching, lead ions

## Abstract

New nanocomposites, Fe_3_O_4_@Au–FITC, were prepared and explored to develop a fluorescent detection of Pb^2+^. The Fe_3_O_4_@AuNPs–FITC nanocomposites could be etched by Pb^2+^ in the presence of Na_2_S_2_O_3_, leading to fluorescence recovery of FITC quenched by Fe_3_O_4_@Au nanocomposites. With the increase of Pb^2+^ concentration, the fluorescence recovery of Fe_3_O_4_@AuNPs–FITC increased gradually. Under optimized conditions, a detection limit of 5.2 nmol/L of Pb^2+^ with a linear range of 0.02–2.0 µmol/L were obtained. The assay demonstrated negligible response to common metal ions. Recoveries of 98.2–106.4% were obtained when this fluorescent method was applied in detecting Pb^2+^ spiked in a lake-water sample. The above results demonstrated the high potential of ion-induced nanomaterial etching in developing robust fluorescent assays.

## 1. Introduction

Heavy metal pollution has been a serious long-term threat to human health and ecosystems due to their toxicity. Lead ions (Pb^2+^) are one example of major, ubiquitous, and bio-accumulative heavy metal pollutants, and can cause damage to the kidneys and brain in various ways, even at low concentration [1,2,3]. The U.S. Environmental Protection Agency (EPA) has set the maximum allowable levels of Pb^2+^ in drinking water at 72 nmol/L (15 ppb) [1].

Conventional analytical methods including atomic absorption spectrometry (AAS), inductively coupled plasma optical emission spectrometry (ICP-OES), inductively coupled plasma mass spectrometry (ICP-MS), etc. are broadly used in central labs for the determination of metal ions [4]. Many methods for the rapid detection of Pb^2+^ were reported including colorimetric [5,6], fluorescent [7,8], and electrochemical methods [9,10]. Among them, the fluorescent method has drawn much attention due to its advantages of high sensitivity, high specificity, and simple operation [11,12,13,14,15].

Nanomaterial-based fluorescent sensors for the detection of Pb^2+^ have attracted a great deal of attention recent years. These methods are mostly related to direct fluorescence quenching or energy transfer processes (i.e., Forster resonance energy transfer (FRET), and nanomaterial surface energy transfer (NSET)). The binding to or recognition of Pb^2+^ mainly includes ion-chelation with ligands [16] or biomolecules (e.g., aptamer, DNAzyme, and antibody) [17] present on the nanomaterial surface.

Another strategy of ion recognition is ion-induced leaching or etching of gold nanoparticles (AuNPs), which have been used to develop non-aggregate AuNPs colorimetric or fluorescent detection for various targets [18,19,20]. Compared with ion-chelation and bio-molecular recognition strategies, very few efforts have been made to develop fluorescent sensing for Pb^2+^ based on the etching of nanomaterials [21].

**Scheme 1:**
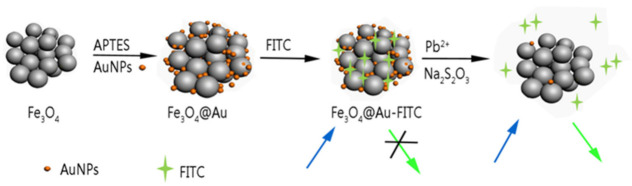
Schematic illustration of Pb^2+^ detection based on Fe_3_O_4_@Au–FITC nanocomposites.

In this work, fluoresce isothiocyanate (FITC) was absorbed onto the surface of Fe_3_O_4_@Au nanocomposites and then the fluorescence of FITC was quenched. The obtained Fe_3_O_4_@AuNPs–FITC nanocomposites could be etched by Pb^2+^ in the presence of Na_2_S_2_O_3_, leading to fluorescence recovery of FITC. Based on this principle, a simple, rapid, and highly sensitive fluorescent detection for Pb^2+^ was established. Scheme 1 shows the principle of Fe_3_O_4_@Au–FITC nanocomposite-based fluorescent detection of Pb^2+^.

## 2. Results

### 2.1. Characterization of NPs

**Figure 1:**
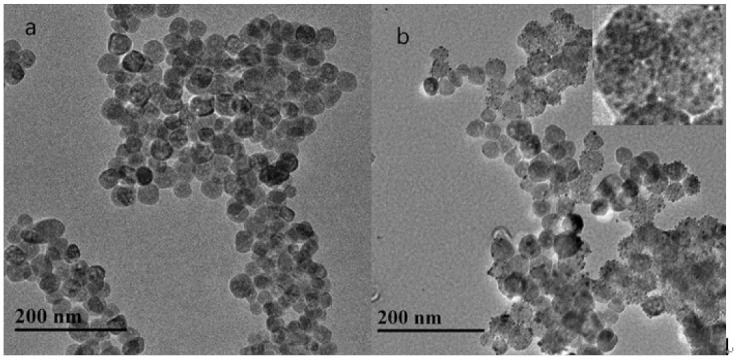
TEM images of (**a**) Fe_3_O_4_ and (**b**) Fe_3_O_4_@Au NPs.

**Figure 2:**
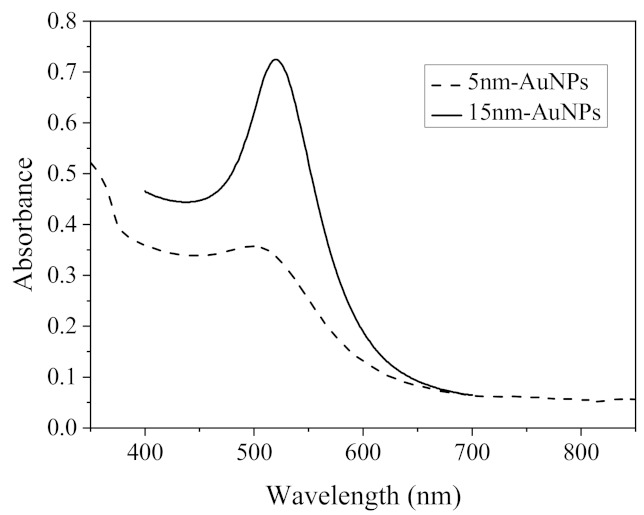
UV-vis spectra of AuNPs.

Figure 1a shows the TEM image of the Fe_3_O_4_, which displays a good dispersion with an average diameter of 30 nm. Since the Fe_3_O_4_ NPs modified with 3-Aminopropyltriethoxysilane (APTES) have abundant amino groups on the surface, the small AuNPs could be absorbed on the Fe_3_O_4_ NPs by the electrostatic interaction between citric acid and amino groups [22]. As shown in (Figure 1b), the AuNPs was dispersed around spherical Fe_3_O_4_ particles with an average diameter of 5.0 nm. The nanocomposites can be easily magnetically separated as shown in Figure S1. The nanocomposites can remain stable after storage for 1 month. As shown in the UV-vis spectra (Figure 2), strong characteristic absorption peaks at 498 nm and 520 nm of small AuNPs (5 nm) and AuNPs (15 nm) can be found [23,24].

### 2.2. Etching of Fe_3_O_4_@AuNPs–FITC by Lead and Thiosulfate Ions

**Figure 3:**
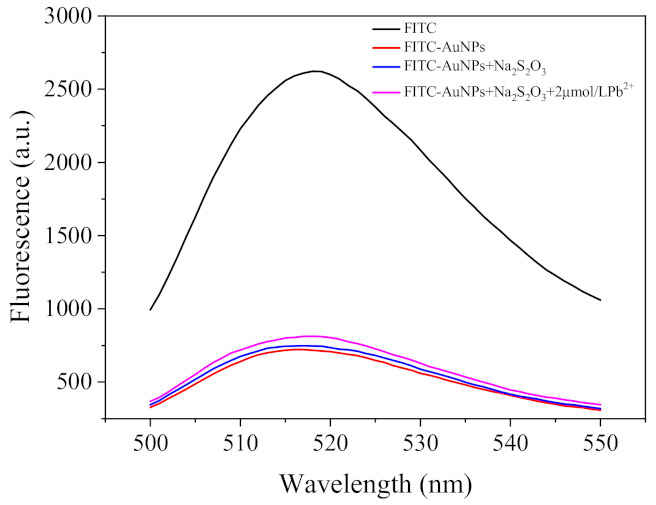
Fluorescence spectra of the FITC–AuNPs.

As shown in Figure 3, the fluorescence emission of FITC was almost completely quenched by AuNPs. With the addition of S_2_O_3_^2−^ and Pb^2+^ ions, the fluorescence intensity of the solution demonstrated slightly increasement. This may be because the above etching of AuNPs (15 nm) only slightly changed the physicochemical property and the etched AuNPs (15 nm) still were big enough to quench FITC.

**Figure 4:**
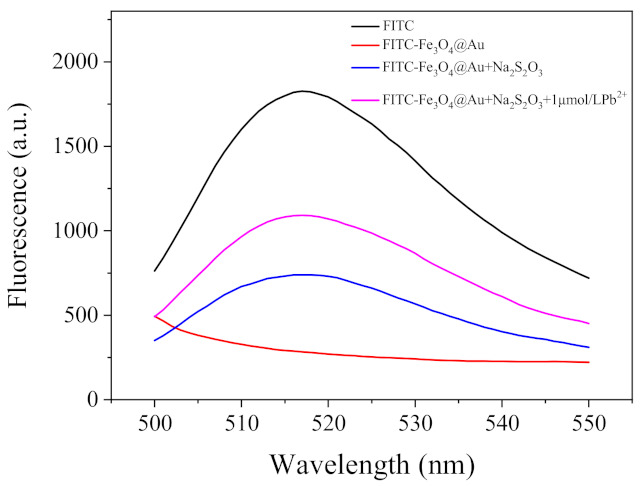
Fluorescence spectra of Fe_3_O_4_@Au–FITC.

Thus, we prepared the Fe_3_O_4_@AuNPs–FITC to further explore whether the strategy of combing the etching of smaller AuNPs, and the AuNPs separation from FITC together has the potential to develop a sensitive sensor. As shown in Figure 4, very weak fluorescent emission is due to the quenching of FITC by AuNPs. In the presence of Na_2_S_2_O_3_, the fluorescent emission of the solution obviously increased. This may be due to the redox reaction between Na_2_S_2_O_3_ and AuNPs in Fe_3_O_4_@AuNPs–FITC, forming the Au(S_2_O_3_)_2_^3+^ compound [25,26] and slightly impairing the adsorption of FITC on the surface of AuNPs. With the coexistence of S_2_O_3_^2−^ and Pb^2+^ ions, the fluorescent emission of the solution increased much more significantly. These results indicate that the etching of AuNPs by ions deserves further investigation for developing a Pb^2+^ sensing method.

### 2.3. Optimization of the Fluorescent Assay

**Figure 5:**
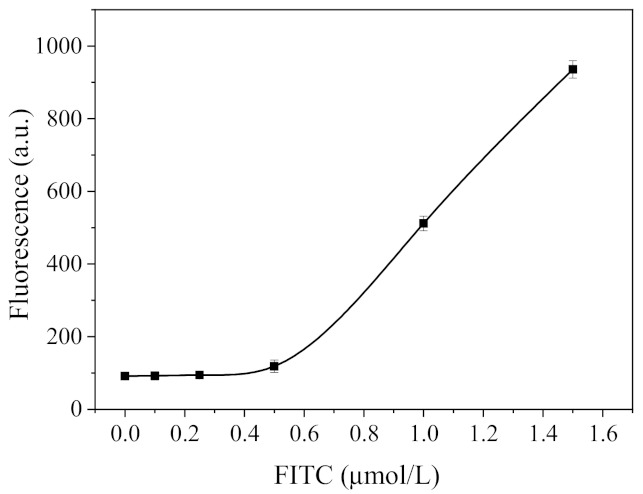
Effect of FITC concentration on Pb^2+^ sensing.

To achieve a sensitive fluorescent assay for Pb^2+^, some factors including FITC concentration, incubation time, Na_2_S_2_O_3_ concentration, and pH value were investigated. As shown in Figure 5, the fluorescence intensity raises when the FITC concentration is higher than 0.5 μmol/L, which indicates excess FITC appears and separates from the Fe_3_O_4_@Au NPs (Figure 5). Thus, 0.5 μmol/L FITC was selected for further experiments.

**Figure 6:**
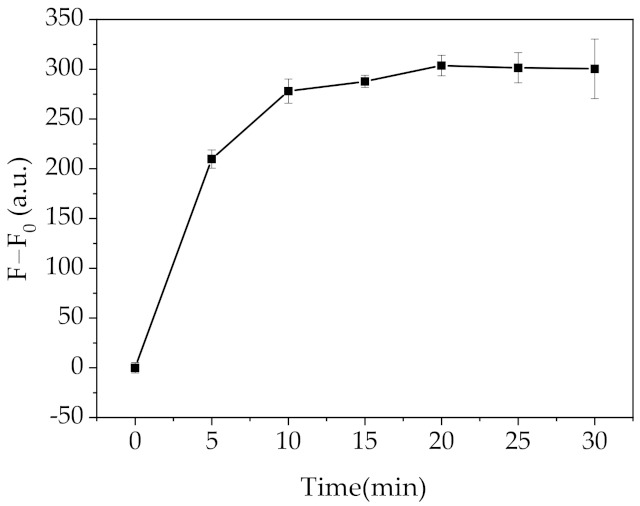
Effect of the incubation time on Pb^2+^ sensing.

As shown in Figure 6, the recovered fluorescence intensity (F-F_0_) increased with the incubation time extension, and nearly leveled off after 10 min. Thus, incubating Fe_3_O_4_@Au–FITC with etching ions for 10 min was enough for sensing Pb^2+^.

**Figure 7:**
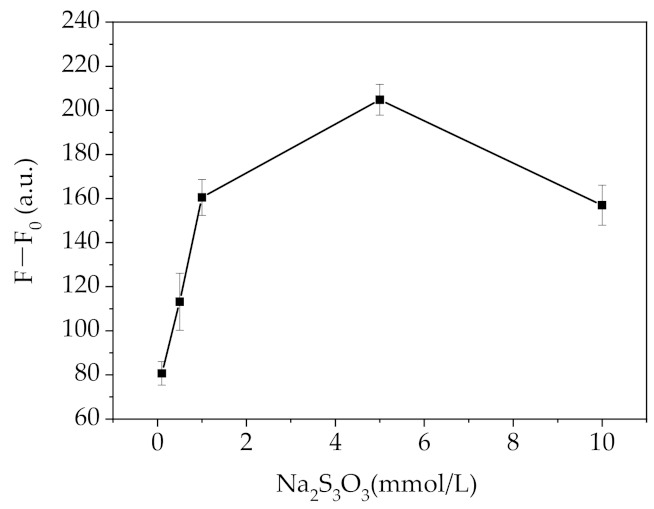
Effect of Na_2_S_2_O_3_ concentration on Pb^2+^ sensing.

As shown in Figure 7, the recovered fluorescence intensity (F-F_0_) displayed a gradual increase in the range 0–5.0 mmol/L Na_2_S_2_O_3_ and a higher concentration decreased the fluorescence signal.

**Figure 8:**
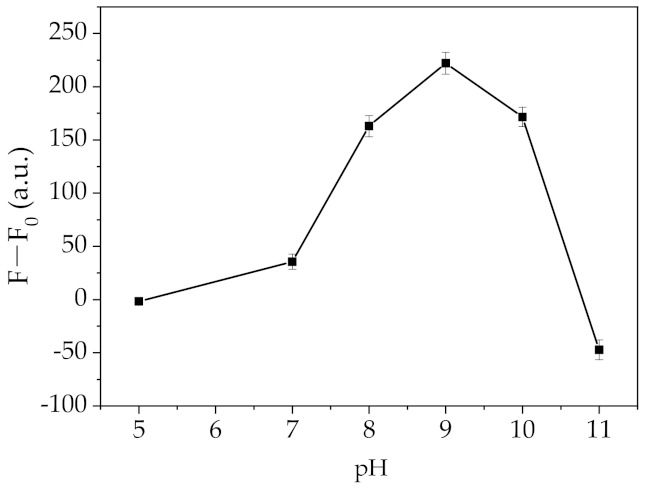
Effect of the pH value on Pb^2+^ sensing.

As shown in Figure 8, the pH value of glycine–NaOH buffer demonstrated big impact on the recovered fluorescence intensity (F-F_0_). However, good fluorescence response could be obtained with the glycine–NaOH buffer at the range of pH 8.0–10 and highest signal obtained at pH 9.0.

### 2.4. Selectivity of Pb^2+^ Sensing

**Figure 9:**
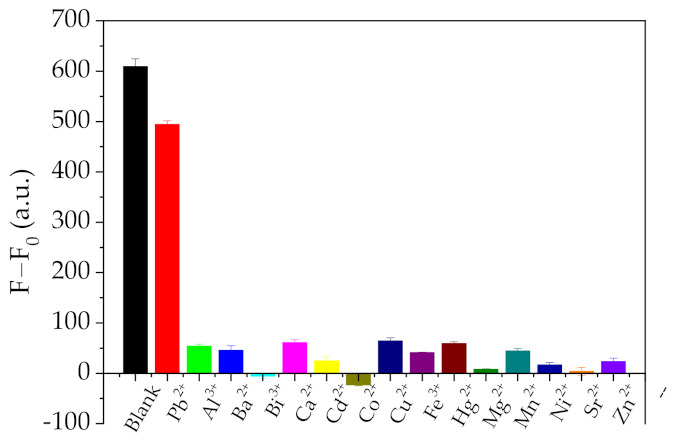
Selectivity of the assay for various metal ions.

The selectivity of the assay was investigated by testing some common metal ions (Cu^2+^, Mg^2+^, Ca^2+^, Co^2+^, Fe^3+^, Sr^2+^, Al^3+^, Pb^2+^, Hg^2+^, Mn^2+^, Cd^2+^, Ni^2+^, Zn^2+^, Ba^2+^, and Bi^2+^, 10 µmol/L) under the same experimental conditions. As shown in Figure 9, all these metal ions produced negligible fluorescence signal to the Fe_3_O_4_@Au–FITC-based assay. The results indicated high selectivity toward Pb^2+^ of this Fe_3_O_4_@Au–FITC-based assay. The high selectivity should be ascribed to two aspects: (1) the magnetic separation and enrichment of Pb^2+^ ions; and (2) the selective etching of AuNPs by Pb^2+^ in the presence of Na_2_S_2_O_3_.

### 2.5. Analytical Performance of Pb^2+^ Sensing

**Figure 10:**
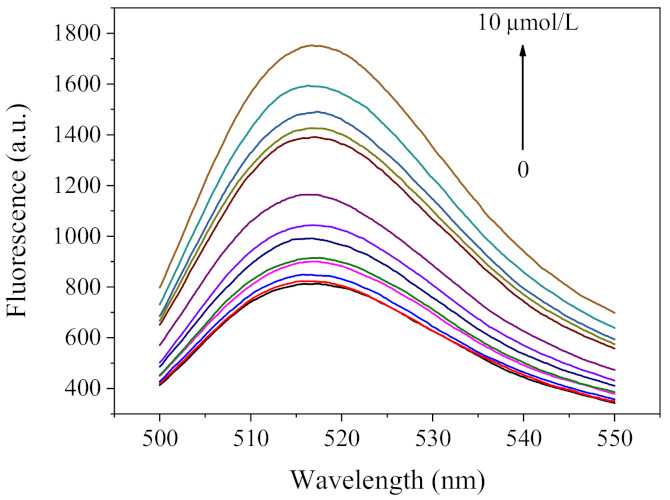
Fluorescence spectra of Fe_3_O_4_@Au–FITC in the presence of various concentrations of Pb^2+^.

**Figure 11:**
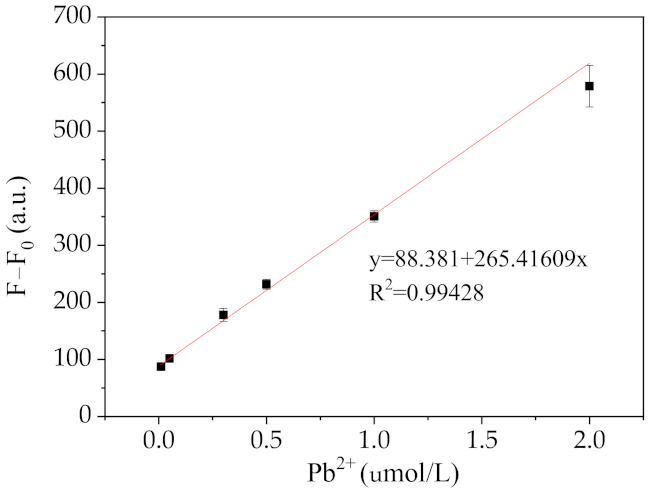
The relationship between the difference value of fluorescence intensities and the different concentrations of Pb^2+^.

Under optimal conditions, the sensitivity of the fluorescence assay was evaluated. As shown in Figure 10, the fluorescence intensity increased gradually with the increasing Pb^2+^ concentration in the range of 0–10 µmol/L. A linear relationship between the fluorescence intensity and Pb^2+^ concentration could be achieved in the range 0.02 to 2.0 µmol/L (Figure 11). The limit of detection (LOD) was 5.2 nmol/L, estimated by a 3-fold signal-to-noise ratio (3S/N). The analytical performance of the Fe_3_O_4_@Au–FITC-based assay was much more sensitive and rapid than the other reported nanomaterial etching-based methods [25,26,27] (Table 1). In addition, the LOD of the proposed method was much lower than the maximum allowable levels of Pb^2+^ (72 nmol/L) in drinking water set by the U.S. EPA.

### 2.6. Application of Pb^2+^ Sensing

Samples collected from Tai lake (Wuxi, China) were filtered through a microfiltration membrane (0.22 μm) and spiked with different concentrations of Pb^2+^ (50, 100 and 150 nmol/L). The samples then were measured by the above fluorescent method. As demonstrated in Table 2, the recoveries from 98.2% to 106.4% were obtained with relative standard deviation (RSD) less than 10%. The above results demonstrated the practical potential of this Fe_3_O_4_@Au–FITC-based fluorescent method for Pb^2+^ in detecting environmental samples.

## 3. Materials and Methods

### 3.1. Chemicals and Reagents

All reagents were of analytical grade and used without further purification. Sodium borohydride (NaBH_4_) and 3-Aminopropyltriethoxysilane (APTES) were purchased from Shanghai Aladdin biochemical technology Co., Ltd. (Shanghai, China) Chloroauric acid hydrate (HAuCl_4_·3H_2_O) and sodium citrate were purchased from Sigma–Aldrich (Shanghai, China). Fluorescein isothiocyanate (isomeride I) (FITC > 95.0%) was purchased from TCI (Shanghai, China) chemical industry Co., Ltd. Copper, aluminum, nickel, cobalt, barium, cadmium, manganese, iron, zinc, strontium, bismuth, chromium were purchased from Guobiao (Beijing, China) Testing & Certification Co., Ltd. The nitrate, acetate, or chloride salts of other metal ions were purchased from Sinopharm Chemical Reagent Co, Ltd.(Shanghai, China). Ethylene glycol, ethanolamine, ethanol and Poly (ethylene glycol) (PEG2000) were purchased from Sinopharm Chemical Reagent Co, Ltd. (Shanghai, China).

### 3.2. Apparatus

UV-visible (UV-vis) absorption spectra of nanoparticles were obtained with a microplate reader (Bio-Tek, Elx800, USA) (Winooski, VT, USA). Steady-state fluorescence spectra were measured by a fluorospectrometric photometer (Lengguan F97, Shanghai Lengguan, China). Transmission electron microscopy (TEM) was performed on a JEOL JEM-2100 (Kyoto, Japan) at an accelerating voltage of 200 kV. Energy diffraction X-ray (EDX) spectrum was obtained using the TEM.

### 3.3. Synthesis of Au Nanoparticles (AuNPs)

AuNPs (5.0 nm) were synthesized by NaBH_4_ reduction method [23]. Briefly, HAuCl_4_ (0.5 mL, 4.0 g/L) and sodium citrate (2.0 mL, 0.025 mmol/L) were dissolved in 20 mL water under magnetic stirring. Then 0.6 mL NaBH_4_ (0.1 mol/L) solution was added and the mixture was stirred thoroughly. The color of the solution changed from light yellow to orange red, which indicated that the AuNPs were successfully synthesized.

### 3.4. Amine-Functionalization of Fe_3_O_4_ Nanoparticles Using APTES

Fe_3_O_4_ nanoparticles were synthesized according to the method with slight modifications [28]. FeCl_3_·6H_2_O (1.05 g), ethylene glycol (21 mL) and ethanolamine (7 mL) were added to the conical flask under magnetic stirring. Then, sodium acetate trihydrate (NaAc) (2.8 g) and PEG2000 (0.7 g) were added to the solution under vigorous stirring. The reaction was maintained at 200 °C for 8 h. Afterwards, the Fe_3_O_4_ NPs were obtained after the mixture was washed several times with water and ethanol. The Fe_3_O_4_ NPs were dried in a vacuum oven at 60 °C for 12 h and Fe_3_O_4_ NPs powder was obtained.

For APTES modification, the Fe_3_O_4_ NPs (150 mg) were suspended in a mixture of ethanol (40 mL) and APTES solution (1.0 mL). The mixture was gently stirred at room temperature for 3 h. The Fe_3_O_4_ NPs were separated using an external magnet, and thoroughly washed with ethanol and water. The amine-functionalization of Fe_3_O_4_ nanoparticles were finally suspended in 15 mL ethanol and stored at 4 °C for subsequent use.

### 3.5. Synthesis of Fe_3_O_4_@AuNCs-FITC

To prepare Fe_3_O_4_@AuNPs, 3.0 mL of amine-functionalization of Fe_3_O_4_ NPs was dissolved in 15 mL of water, and then 100 mL of AuNPs (5 nm) was added under stirring for 15 min. The Fe_3_O_4_@AuNPs were separated using an external magnet, washed with water and finally suspended in 25 mL of water [29]. The Fe_3_O_4_@AuNP-FITC nanocomposites were synthesized as follows. FITC (50 µL,50 µmol/L) onto Fe_3_O_4_@Au NPs (250 µL) in 4.7 mL water and stored at 4 °C overnight. FITC–AuNPs was prepared as follows. AuNPs (15 nm) were prepared using trisodium citrate reduction method [24]. Briefly, 100 mL of 0.01% HAuCl_4_ solution was slowly stirred, heated to a boil in the conical flask and kept boiling for 5 min. Then, 2 mL of 1% trisodium citrate solution was poured into the flask. The color of mixture changed from pale yellow to gray and finally to wine red. After boiling for 10 min, the obtained AuNPs (3.0 nmol/L) were cooled to room temperature and stored at 4 °C for subsequent use. FITC–AuNPs nanocomposites were synthesized according to the method reported by Wang et al. [30]. FITC solution (5.0 µL, 1.5 mmol/L) in ethanol was added into 5.0 mL of AuNPs solution and mixed gently. The mixture was stored at 4 °C overnight and then FITC–AuNPs nanocomposites were obtained.

### 3.6. Fluorescent Detection of Pb^2+^

A total of 10 µL of Fe_3_O_4_@AuNP–FITC composites, 40 µL of H_2_O, and 50 µL of sodium thiosulfate (20 mmol/L) as well as 50 µL of glycine–NaOH buffer (10 mmol/L, pH 9.0) were mixed gently. After incubation at room temperature for 5.0 min, Pb^2+^ solution was added and incubated at room temperature for 10 min. Then the Fe_3_O_4_@AuNPs were separated by an external magnet for 2 min. The fluorescence emission spectra of the supernatant were measured by a fluorescence spectrometer with 465 nm excitation.

## 4. Conclusions

A highly sensitive and selective fluorescent method for Pb^2+^ sensing was developed based on a new nanocomposite, Fe_3_O_4_@Au–FITC. This method combined the metal leaching by the Pb^2+^-S_2_O_3_^2−^ system with magnetic separation together. Considering that ion-induced etching of metal NPs has been applied in the development of analytical methods for the detection of metal ions, anions, small molecules, and proteins [31,32], the strategy proposed here is expected to be further explored in some challenging situations.

## Figures and Tables

**Table 1:** Comparison of several methods for Pb^2+^ detection.

Nanoprobes	Linear Range	LOD	Time	Ref.
CTAB modified AuNPs	1.0~6.0 µmol/L	75 nmol/L	30 min	[25]
AuNPs and graphene oxide	0.1~20 µmol/L	50 nmol/L	20 min	[27]
AuNPs	0.0025~10 µmol/L	0.5 nmol/L	2 h	[26]
Fe3O4@Au–FITC	0.02~2.0 µmol/L	5.2 nmol/L	12 min	This work

**Table 2:** Determination of Pb^2+^ in lake-water samples (n = 3).

Concentration (nmol/L)	Result (nmol/L)	Recovery (%)	RSD (%)
50	53.2	106.4	8.3
100	98.3	98.3	2.5
150	159.0	106.0	3.7

## Data Availability

Not applicable.

## Supplementary Materials

The following are available online, Figure S1: Magnetic seperation of the Fe_3_O_4_@Au–FITC nanocomposites.
