# Supplementary figures and images for: Highly Sensitive and Selective Fluorescence “Turn-On” Detection of Pb (II) Based on Fe3O4@Au–FITC Nanocomposite
Source: Molecules. 2021 May 26;26(11):3180. doi: 10.3390/molecules26113180 (PMC8198146; doi:10.3390/molecules26113180)

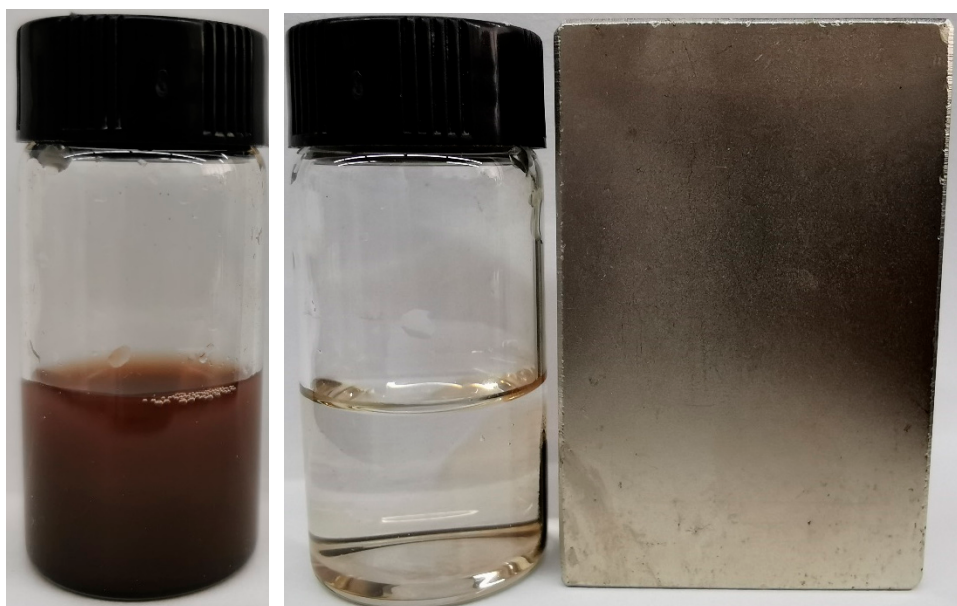

**Figure S1.** Magnetic separation of the  $\text{Fe}_3\text{O}_4@\text{Au}$ -FITC nanocomposites.

Supplement: Supplementary file 1 [file molecules-26-03180-s001.zip › molecules-1206610-SI.pdf]
